# Supplementary material for: Clostridioides (Clostridium) difficile infection in hospitalized adult patients in Cambodia
Source: Microbiol Spectr. 2025 Feb 19;13(4):e02747-24. doi: 10.1128/spectrum.02747-24 (PMC11960136; doi:10.1128/spectrum.02747-24)
Supplement: Supplemental material — Tables S1 and S2. [file spectrum.02747-24-s0001.pdf]

SUPPLEMENTAL TABLES

Table S1 Socio-demographic, clinical and other features.

Socio-demographic factor	Number of cases	
	**Without *C. difficile* (N = 200) n**	**With *C. difficile* (N = 63) n**
Sex (female)		
Yes	111	32
No	89	30
Age (IQR: 44 – 68)		
≥65 years	64	25
<65 years	135	37
Residency: Living outside the capital city		
Yes	143	51
No	47	7
Living close to livestock		
Yes	56	14
No	139	48
Live with siblings < 1 year old		
Yes	7	2
No	193	60
**Clinical and other feature**		
Blood leucocyte > 9Giga/L		
Yes	83	28
No	89	30
Stool consistency (loose)		
Yes	19	7
No	178	56
Diarrhoea		
Yes	28	5
No	170	57
Abdominal pain		
Yes	44	22
No	156	41
Fever		
Yes	58	23
No	141	40
Hepatitis/cirrhosis		
Yes	16	3
No	184	60
Anaemia and other blood disorders		
Yes	15	2
No	185	61
Tuberculosis		
Yes	4	1
No	196	62
Pneumonia/ chronic obstructive pulmonary disease		
Yes	54	18
No	146	45
Acute pneumonia oedema		
Yes	9	3
No	191	60
Other respiratory/lung conditions		
Yes	23	2
No	177	61
Thyroid diseases		
Yes	7	0
No	193	63
Chest pain		
Yes	2	0
No	198	63
Lower limb oedema		
Yes	1	1
No	199	62
Pancreatitis		
Yes	5	1
No	195	62
Nephrotic syndrome		
Yes	1	2
No	199	61

Urinary tract infection		
Yes	17	3
No	193	60
Other bacterial infections		
Yes	9	5
No	191	58
Sepsis		
Yes	3	1
No	197	62
Dengue		
Yes	2	1
No	198	62
Mycosis		
Yes	1	1
No	199	62
Gastritis		
Yes	2	1
No	198	62
Digestive haemorrhage		
Yes	2	0
No	198	63
Cholecystitis		
Yes	3	3
No	197	60
Cardiopathy		
Yes	7	4
No	193	59
Heart failure		
Yes	24	12
No	176	51
Polyarthritis		
Yes	7	1
No	193	62
Bowel function disorders		
Yes	2	3
No	198	60
Oesophageal variceal bleeding		
Yes	1	3
No	199	60
Lupus		
Yes	2	1
No	198	62
Diabetes		
Yes	65	30
No	135	33
Cortico-adrenal insufficiency		
Yes	9	4
No	191	59
Renal diseases		
Yes	33	16
No	167	47
Hypertension		
Yes	67	28
No	133	35
Gout		
Yes	2	0
No	198	63
HIV		
Yes	3	1
No	197	62
Stroke		
Yes	8	2
No	191	61
Haematological malignancy		
Yes	2	0
No	197	63
Cancer		
Yes	12	4
No	185	59
Frequent outpatient department (OPD) visits (at least 1/week)		
Yes	19	13
No	178	50
Non-surgical gastrointestinal procedure		
Yes	40	16
No	158	47

Gastrostomy		
Yes	2	0
No	194	63
Haemodialysis		
Yes	2	4
No	196	59
Surgery		
Yes	27	12
No	169	51
**1 week prior to stool collection**		
Gastrostomy		
Yes	1	0
No	195	63
Haemodialysis		
Yes	0	2
No	196	61
Surgery		
Yes	2	1
No	187	60
**4 weeks prior to stool collection**		
Gastrostomy		
Yes	0	0
No	199	63
Haemodialysis		
Yes	0	0
No	198	63
Surgery		
Yes	0	1
No	182	61
**3 months prior to stool collection**		
Hospitalisation >24 h		
Yes	46	25
No	148	37
Frequent OPD visits (at least 1/week)		
Yes	15	9
No	183	53
Reside in a long-term care facility		
Yes	8	3
No	188	59

Table S2 Consumption of antimicrobials and other medications.

Antimicrobial and other medication	Number of cases	
	**Without *C. difficile* (N = 200)**	**With *C. difficile* (N = 63)**
	**n**	**n**
Metronidazole		
Yes	30	9
No	169	54
Vancomycin		
Yes	3	2
No	195	61
Amoxicillin + clavulanic acid		
Yes	13	2
No	187	61
Cefoperazone + sulbactam		
Yes	3	1
No	197	62
Piperacillin + tazobactam		
Yes	33	14
No	167	49
Third-generation cephalosporins		
Yes	39	13
No	161	50
Carbapenems		
Yes	34	12
No	166	51
Fluoroquinolones		
Yes	39	9
No	161	54
Amikacin		
Yes	4	0
No	196	63
Azithromycin		
Yes	11	2
No	189	61
Sulfamethoxazole + trimethoprim		
Yes	9	2
No	191	61
Doxycycline		
Yes	3	0
No	197	63
Linezolid		
Yes	2	1
No	198	62
Antifungal agents		
Yes	1	0
No	199	63
Tuberculosis treatment		
Yes	3	1
No	197	62
Antiparasitic agents		
Yes	13	2
No	185	61
Chemotherapy		
Yes	6	1
No	190	62
Corticosteroids		
Yes	50	16
No	147	47
Immunosuppressants		
Yes	3	1
No	193	62
Proton pump inhibitors		
Yes	174	61
No	23	1
H2 receptor antagonists		
Yes	10	0
No	188	63
Probiotics		
Yes	3	1
No	195	62
Statins		
Yes	15	5
No	182	58
Other medications		
Yes	97	34
No	102	29

**1 week prior to stool collection**		
Antimicrobial consumption		
Yes	56	25
No	123	34
Metronidazole		
Yes	18	1
No	160	55
Vancomycin		
Yes	2	1
No	178	54
Antiparasitic agents		
Yes	2	0
No	187	62
Chemotherapy		
Yes	6	0
No	191	63
Corticosteroids		
Yes	24	9
No	170	51
Immunosuppressants		
Yes	1	0
No	192	61
Proton pump inhibitors		
Yes	75	31
No	115	32
H2 receptor antagonists		
Yes	6	0
No	187	60
Probiotics		
Yes	0	0
No	196	6
Statins		
Yes	7	3
No	186	57
Other medications		
Yes	74	27
No	110	34
**4 weeks prior to stool collection**		
Antimicrobial consumption		
Yes	6	5
No	169	51
Metronidazole		
Yes	1	0
No	175	55
Vancomycin		
Yes	0	1
No	180	56
Antiparasitic agents		
Yes	0	0
No	191	61
Chemotherapy		
Yes	3	0
No	191	63
Corticosteroids		
Yes	9	2
No	178	59
Immunosuppressants		
Yes	0	0
No	196	60
Proton pump inhibitors		
Yes	22	5
No	165	55
H2 receptor antagonists		
Yes	1	0
No	190	60
Probiotics		
Yes	0	0
No	196	61
Statins		
Yes	7	0
No	189	60
Other medications		
Yes	49	19
No	130	42
